# Aerosol generation and control in the dental operatory: An *in vitro* spectrometric study of typical clinical setups

**DOI:** 10.1371/journal.pone.0246543

**Published:** 2021-02-04

**Authors:** Fruzsina Kun-Szabó, Dorottya Gheorghita, Tibor Ajtai, Szabolcs Hodovány, Zoltán Bozóki, Gábor Braunitzer, Márk Ádám Antal

**Affiliations:** 1 Department of Optics and Quantum Electronics, Faculty of Science and Informatics, University of Szeged, Szeged, Hungary; 2 Department of Esthetic and Operative Dentistry, Faculty of Dentistry, University of Szeged, Szeged, Hungary; 3 Department of Research, dicomLAB Dental Ltd., Szeged, Hungary; University of Shanghai for Science and Technology, CHINA

## Abstract

Dental turbines and scalers, used every day in dental operatories, feature built-in water spray that generates considerable amounts of water aerosol. The problem is that it is not exactly known how much. Since the outbreak of COVID-19, several aerosol safety recommendations have been issued—based on little empirical evidence, as almost no data are available on the exact aerosol concentrations generated during dental treatment. Similarly, little is known about the differences in the efficacy of different commercially available aerosol control systems to reduce in-treatment aerosol load. In this *in vitro* study, we used spectrometry to explore these questions. The time-dependent effect of conventional airing on aerosol concentrations was also studied. Everyday patient treatment situations were modeled. The test setups were defined by the applied instrument and its spray direction (high-speed turbine with direct/indirect airspray or ultrasonic scaler with indirect airspray) and the applied aerosol control system (the conventional high-volume evacuator or a lately introduced aerosol exhaustor). Two parameters were analyzed: total number concentration in the entire measurement range of the spectrometer and total number concentration within the 60 to 384 nm range. The results suggest that instrument type and spray direction significantly influence the resulting aerosol concentrations. Aerosol generation by the ultrasonic scaler is easily controlled. As for the high-speed turbine, the efficiency of control might depend on how exactly the instrument is used during a treatment. The results suggest that scenarios where the airspray is frequently directed toward the air of the operatory are the most difficult to control. The tested control systems did not differ in their efficiency, but the study could not provide conclusive results in this respect. With conventional airing through windows with a standard fan, a safety airing period of at least 15 minutes between treatments is recommended.

## Introduction

Dental procedures are especially risky in terms of aerosol production [1–3]. Aerosol control in the dental setting is not an entirely new topic, but the COVID-19 epidemic has obviously boosted interest in it. Earlier studies dealt with questions like spatter reduction during ultrasonic scaling [4], the reduction of the bacterial contamination of the aerosol itself [5] and reducing staff exposure to ultrafine particles from the surface sprays used for optical scanning with high-volume evacuator (HVE) [6]. While HVE proved to be highly effective in the latter case, Desarda and colleagues concluded that HVE, when used in itself, is not effective in reducing aerosol counts and environmental contamination [7]. On the contrary, Devker et al. concluded that HVE, either alone or combined with chlorhexidine rinsing is effective in reducing the mean colony forming bacterial units in aerosol produced during scaling [8].

After the first wave of the pandemic, dental professionals are introducing new protective and preventive protocols for the daily practice. The virus may attach to aerosol particles of various sizes, resulting in combined particle sizes from 60 to 300 nm [9]. Aerosol particles under 10 μm can penetrate even the smallest airways in the lungs [10]. While the expected performance of filtering facepieces (FFPs) is regulated in standards, relatively little is known about their real-life performance. The only reliable study dealing with this question is that of Lee and colleagues, who found that FFP 1–3 masks protected invariably well against particles in the 93–1610 nm range but found a range of (not significantly) weaker protection between 263 and 384 nm [11]. Thus, while they perform well, even FFP masks cannot offer complete protection. It is already known that SARS-CoV-2 can remain viable and infectious in aerosols for up to 3 hours [12], so to work with open windows and keep 10 to 15-minute periods of airing between patients are often seen recommendations [13]. Regarding air conditioning units, recommendations range from not to use them at all to use but sanitize frequently [14].

The main source of aerosol production in the dental setting is the water spray of dental turbines and ultrasonic scalers [15]. The particles in the spray spread either directly from the nozzle or reflected from various surfaces, including the patient’s intraoral hard and soft tissues. It is expected that different kinds of dental treatment are associated with different patterns of aerosol production, depending on the instrument used and the way it is used.

It was foreseeable that the pandemic would boost the need for aerosol control in dentistry, but in lack of empirical data on what concentrations of aerosol are generated during a treatment and how effectively aerosol concentration is reduced by aerosol control systems, it is difficult to give evidence-based recommendations. The available recommendations (mostly from before the outbreak) fail to offer more than emphasizing the importance of aerosol control. For instance, CDC has recommended the use of HVE for long, but it is not supported by actual measurements and no comparison is offered with other systems [16–18]. Of course, before COVID-19, aerosol generation did not seem to be a crucial issue, even if its importance was recognized. However, the new situation demands a different approach, especially that new aerosol control systems are appearing on the market.

In this *in vitro* study, we sought to model typical treatment setups to find out about aerosol production and aerosol control in a clinically relevant manner. The setups were defined by the instrument (high-speed turbine with airspray or ultrasonic scaler with airspray) and the applied aerosol control system (the conventional high-volume evacuator or a lately introduced aerosol exhaustor). The turbine and the ultrasonic scaler are used differently: when used correctly, the water spray from the ultrasonic scaler always hits the teeth first (i.e. aerosol never gets directly in the air). In contrast, the turbine is moved around in all directions during a treatment, so aerosol spreads both directly and indirectly. Thus, for the turbine measurements, we differentiated between direct and indirect spray directions. We hypothesized that both the instrument/spray direction and the aerosol control system would be significant determinants of aerosol concentration.

The effect of post-treatment airing on aerosol concentrations was also studied for each setup. Regarding the effect of airing, we hypothesized that a regular method of airing manageable in any dental operatory would be sufficient to reduce aerosol concentration in a clinically reasonable timeframe between two treatments.

## Materials and methods

### Experimental design

**Fig 1:**
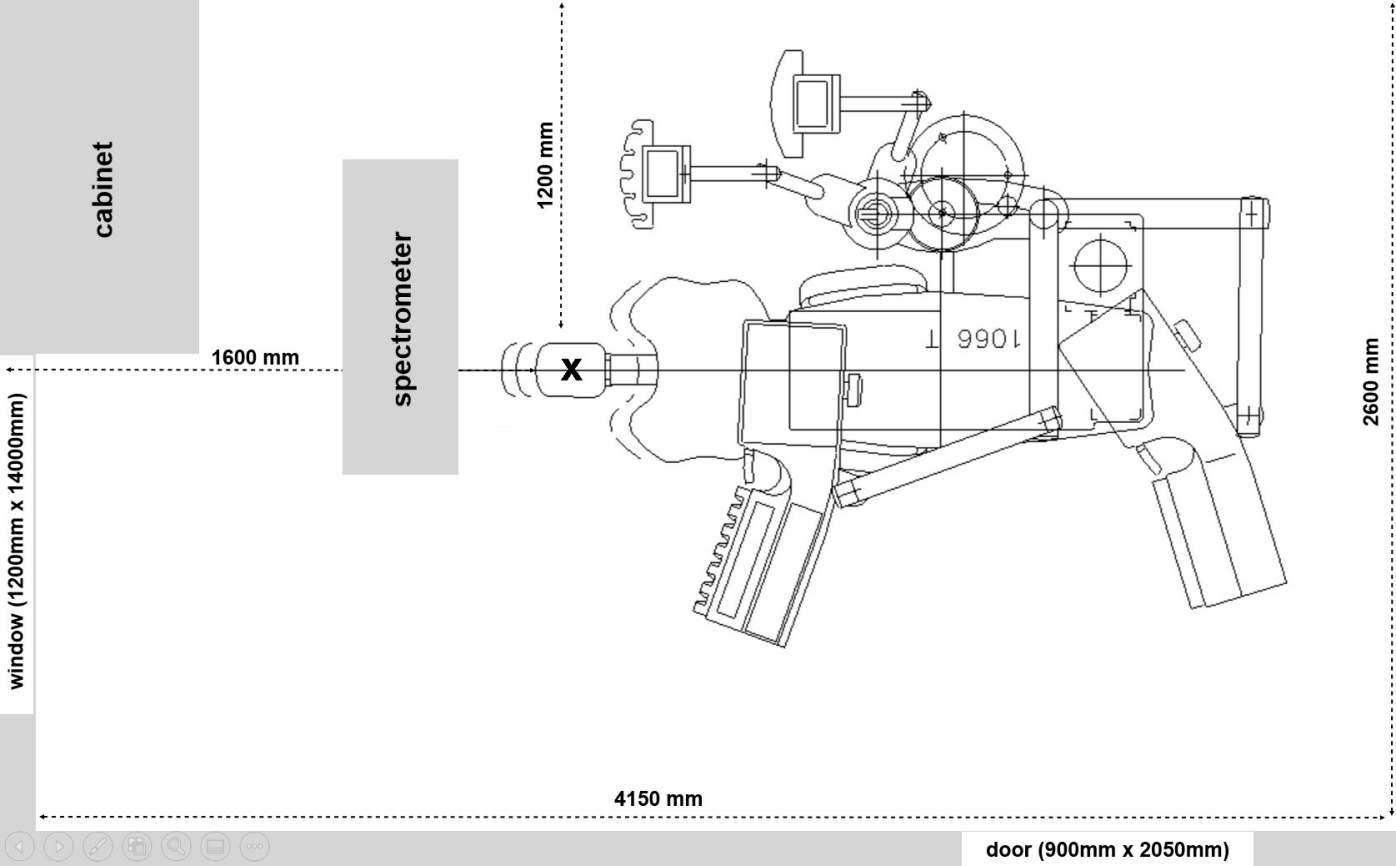
Setup of the site. X marks the position of the mannequin head. The spectrometer was placed on a 935 mm high table, so its sampling tube was 970 mm above the floor of the room. At this height, the sampling tube was 20 cm over the mannequin head. The dimensions of the door and window are given as width x height.

An experimental setup was prepared in a regular dental operatory (4.15 m x 2.6 m) with one door and one window (Fig 1).

**Fig 2:**
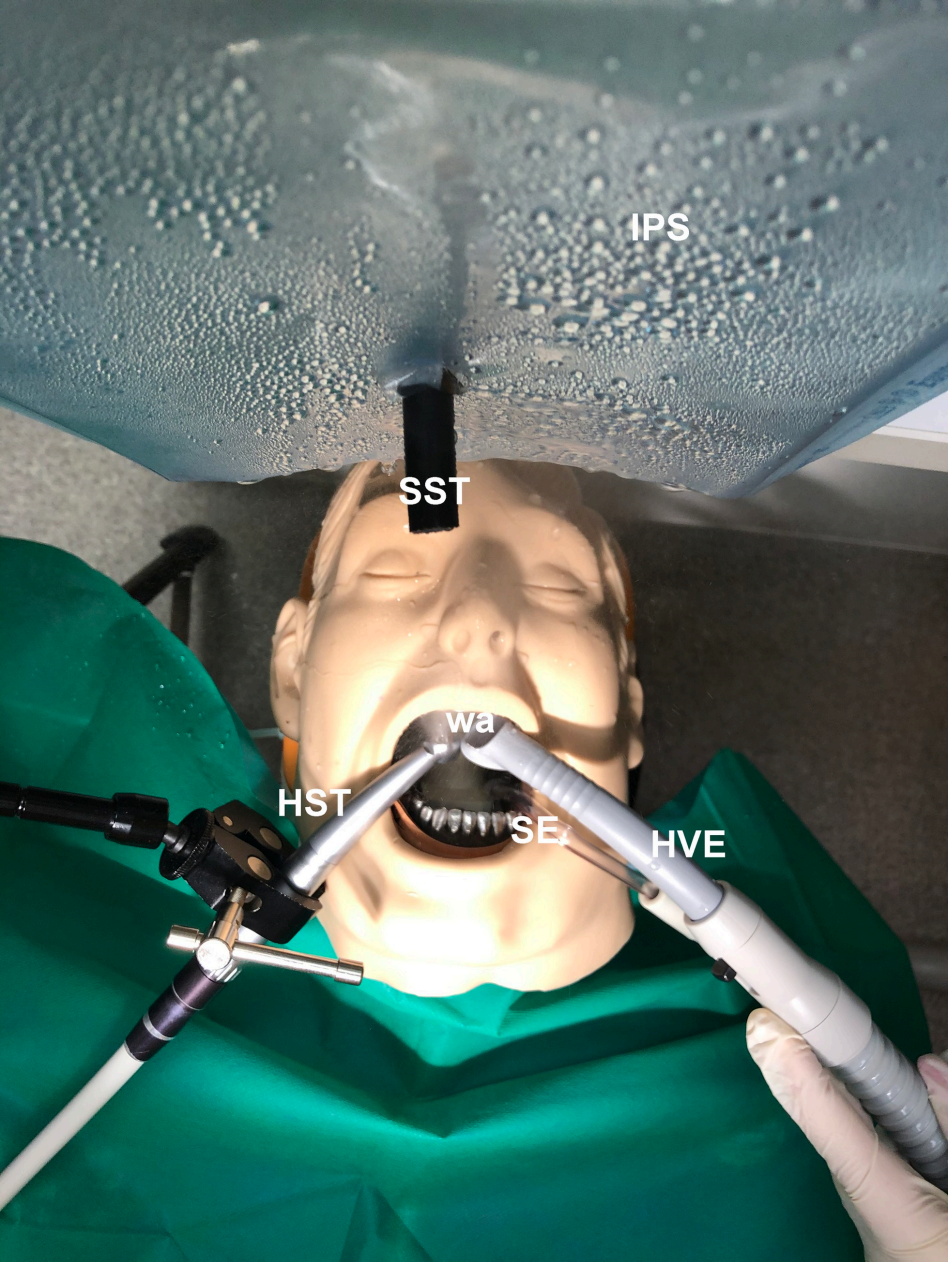
Close-up of the experimental setup. The figure shows the direct spray-high volume evacuator setup (for the rest of the setups, see Fig 3). HST: high-speed turbine; SE: saliva ejector; HVE: high volume evacuator; SST: sampling tube of the spectrometer; IPS: instrument protecting shield (plastic shield to protect the spectrometer from the produced water aerosol); wa: water aerosol generated by the turbine.

To simulate a patient, a mannequin head was used in the supine position. The turbine (Gentle Silence, KaVo Dental, Germany) or ultrasonic scaler (Woodpecker UDS K-LED, Woodpecker, China) was attached to a holder, which allowed to fasten the instrument in a fixed and reproducible position. The high-volume evacuator (N1, Dürr Dental, Germany) or aerosol exhaustor (DentArt Technik, Hungary) were attached to the same dental unit (KaVo 1066 T, KaVo Dental, Germany) and positioned according to the manufacturer’s instructions. According to the literature, the working distance in dentistry falls between 25–33 cm [19]. As protective equipment (such as a face shield) can compromise vision, this is reduced when working in such equipment, thus maximum aerosol load was measured at 20 cm from the mannequin head. Measurements were carried out with a Scanning Mobility Particle Sizer (SMPS-3938) spectrometer (TSI, Minnesota, USA). The endpiece of the spectrometer was positioned above the head of the mannequin at this distance (Fig 2).

### Test measurements

All measurements were carried out in one day. Before the test measurements, the operatory had been intensively aired and air purified (AC3256/20, Philips, Eindhoven, Netherlands) for 12 hours. This was followed by baseline aerosol determination and then the measurements for the different setups. Aerosol reduction was repeated after each test measurement by airing (see below).

**Fig 3:**
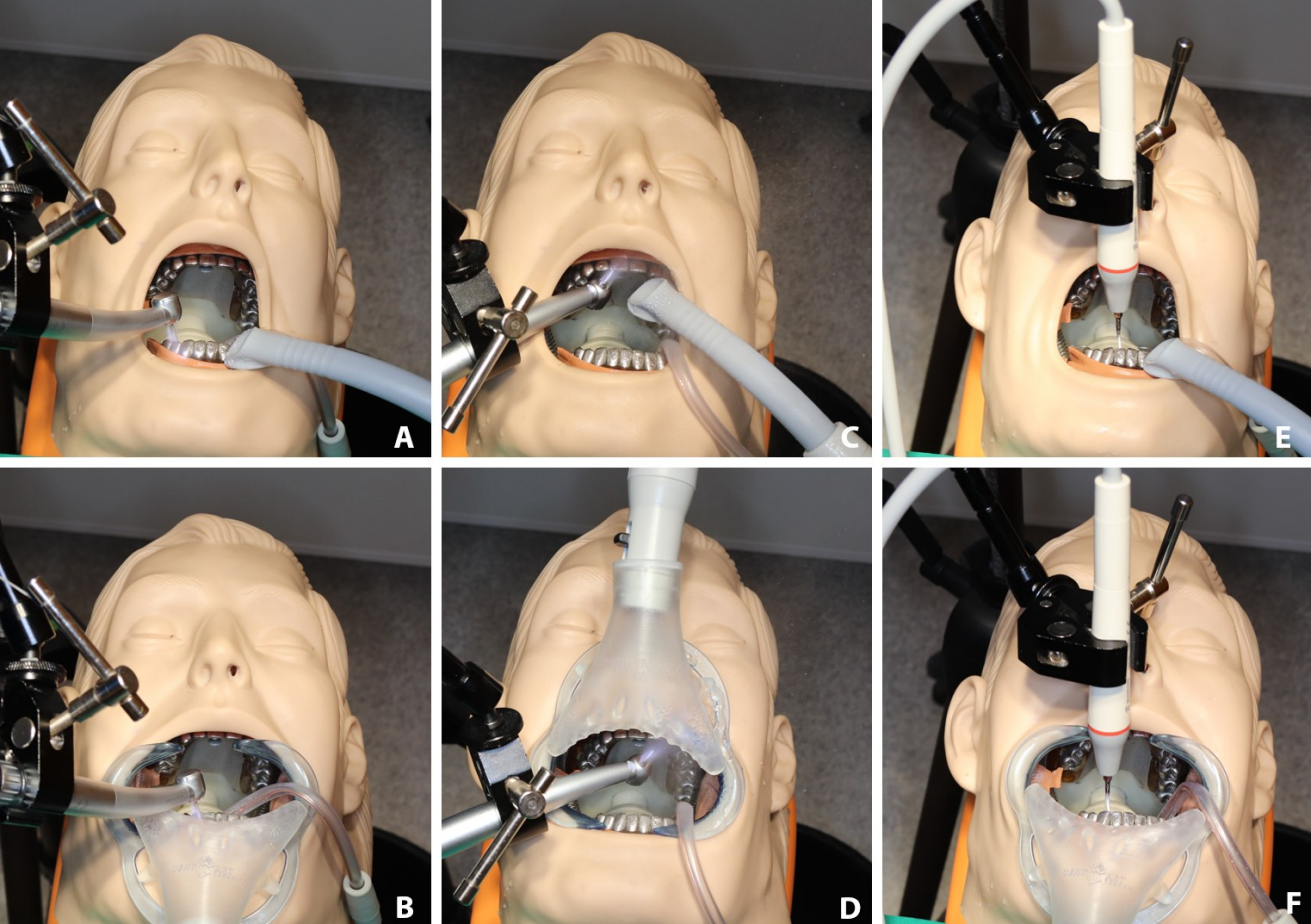
Setups for the modeling of the different clinical scenarios. A) indirect spray with high volume evacuator (IS-HVE), B) indirect spray with aerosol exhaustor (IS-AE), C) direct spray with high volume evacuator (DS-HVE), D) direct spray with aerosol exhaustor (DS-AE), E) ultrasonic scaler with high volume evacuator (US-HVE), F) ultrasonic scaler with aerosol exhaustor (US-AE).

We tested the following setups: a) turbine, direct spray, high-volume evacuator (DS-HVE); b) turbine, indirect spray, high-volume evacuator (IS-HVE); c) turbine, direct spray, aerosol exhaustor (DS-AE); d) turbine, indirect spray, aerosol exhaustor (IS-AE); e) ultrasonic scaler, high-volume evacuator (US-HVE); f) ultrasonic scaler, aerosol exhaustor (US-AE). Fig 3 shows the experimental settings for these setups.

In the direct condition, the turbine faced the palatal surface of the maxillary front teeth allowing the spray to spread directly toward the spectrometer. In the indirect condition, the turbine faced the buccal surface of the mandibular front teeth, so the spray hit the teeth first and then spread indirectly toward the spectrometer. The measurements for each setup were carried out in triplicate, lasted 1 measurement cycle (326 s), and were separated by airing for 3 measurement cycles, during which concentration decay was measured. Values from all three measurements were used for the analyses.

Airing was done by opening both the door and the window of the operatory, while operating a standard fan directed toward the window.

### Statistical analysis

Two parameters were recorded and analyzed: total number concentration for the entire measurement range of the instrument, that is 1.02–982.2 nm (*TNC*: the total number of particles/cm^3^) and total number concentration within the range 60 nm—384 nm (*TNC 60–384*: the number of particles in the 60–384 nm range/cm^3^). This latter range was defined as the combination of the results of Leung et al [9] regarding COVID-relevant aerosol particle sizes (60–300 nm), and the particle size range of weaker FFP protection (263–384 nm) described by Lee and colleagues [11]. The resulting range, according to our present knowledge, is relevant both in terms of COVID and the known relative deficits of FFP mask protection. Aerosol control was defined as the degree to which a given aerosol control system managed to keep water aerosol concentrations close to the baseline in any given setup. Aerosol control for any given setup was expressed as the magnitude of the difference between mean baseline concentration and the mean concentration generated during the measurement cycles for the given setup.

Statistical analyses were performed in SPSS 23.0 (IBM, USA). Continuous variables were described as means, medians, standard deviations, minima, and maxima. Multiple linear regression analysis was used with instrument/spray direction (IS/DS/US) and aerosol control system (HVE/AE) as the independent variables for both *TNC* and *TNC 60–384*, to determine their relative contributions to the variance of the values of the parameters as dependent variables. Pairwise comparisons both in comparisons to the baseline and across-setups comparisons were performed with the t-test (two-tailed). Because of the multiple comparisons, the limit of significance was lowered to *p* = 0.008.

## Results

### Aerosol control: The baseline and deviations from it

After 12 hours of airing, the following values were measured: *TNC—*696.6±94.3/cm^3^; *TNC 60–384*–243.3±28.1/cm^3^. These were considered as the baseline or background values.

The only setup where no significant difference from the baseline was found was US-HVE. Table 1 shows that neither of the measured parameters rose above or sunk below the baseline to a statistically significant extent in this setup. It was in this setup that the values remained the closest to the baseline.

**Table 1:** The results of the measurements.

SETUP	*TNC*	*TNC 60–384*
	(mean ±SD, 1/cm^3^)	(mean ±SD, 1/cm^3^)
**IS-HVE**	626.4±87.1	351.5±24.2
**IS-AE**	1951.1±120.5	864.5±136.7
**DS-HVE**	8530.5±1639	4557.9±2575.5
**DS-AE**	4742.3±407.1	2189.5±174.6
**US-HVE**	621.3±249.4	240.4±76.0
**US-AE**	509.8±27.9	188.1±25.8
**Baseline**	696.6±94.3	243.3±28.1

*TNC*: total number count, *TNC 60–384*: total number count within the range 60 nm– 384 nm. Conventions regarding the study setups are the same as in Figs 1 and 2. Baseline: values measured at the beginning of the day, after 12 hours’ airing. Means and standard deviations in each group come from 3 consecutive measurements (N = 3, see Test Measurements).

As for the rest of the setups: in IS-HVE, only *TNC 60–384* (t = -5.06, df = 4, p = 0.007) was significantly elevated as compared to the baseline.

In IS-AE, significant elevation was observed for both study parameters, as follows: *TNC* (t = -14.19, df = 4 p< 0.001), *TNC 60–384* (t = -7.70, df = 4 p = 0.002)

In DS-HVE, *TNC* (t = -8.26, df = 4 p = 0.001) was significantly higher than the baseline, but *TNC 60–384* (t = -2.9, df = 4 p = 0.044) was not. It must be seen, though, that the latter was still a considerable difference.

In DS-AE, similarly to IS-AE, both study parameters were significantly elevated in comparison to the baseline: *TNC* (t = -16.77, df = 4 p< 0.001), *TNC 60–384* (t = -19.06, df = 4 p< 0.001).

In US-HVE, both parameters showed a decreasing tendency, but it was not significant: *TNC* (t = 0.49, df = 4 p = 0.651), *TNC 60–384* (t = 0.06, df = 4 p = 0.953).

Finally, in US-AE, similarly to US-HVE, both parameters showed a decreasing tendency, which was not statistically significant: *TNC* (t = 3.286, df = 4 p = 0.03), *TNC 60–384* (t = 2.509, df = 4 p = 0.07).

As shown in Table 1, the outcome variables showed elevation compared to the baseline in all setups, which was almost always statistically significant, except for *TNC 60–384* in DS-HVE. In US-HVE and US-AE, decreasing tendencies were observed, but these were not statistically significant for any of the variables. All in all, in terms of aerosol control, the most well-controlled setups were US-AE and US-HVE, followed by IS-HVE, IS-AE, DS-AE and DS-HVE, the latter being the least efficient.

From this, we inferred that the applied instrument/spray direction (DS/IS/US) was of primary importance in terms of aerosol control. To test this hypothesis, multiple linear regression analysis was conducted for both study parameters to determine the relative contributions of the applied instrument/spray direction (DS/IS/US) and the applied aerosol control system (HVE/AE) to the variance of *TNC and TNC 60–384*. The results indicated that the model was a significant predictor of *TNC* (F(2,15) = 17.75, p<0.001, R^2^ = 0.70. Instrument/spray direction contributed significantly to the model (β = -0.83, p< 0.001), but aerosol control did not (β = 0.143, p = 0.326). The model was also a significant predictor of *TNC 60–384* (F(2,15) = 9.18, p<0.01, R^2^ = 0.55. Instrument/spray direction contributed significantly to the model (β = -0.72, p< 0.01), but aerosol control did not (β = 0.178, p = 0.321). Aerosol control alone did not contribute significantly to the variance of either parameter. Thus, all further analyses were done within the groups defined by instrument and spray direction.

### Aerosol control: Comparisons within the groups defined by instrument and spray direction

The comparisons brought the following results: IS-AE was characterized by significantly higher values in both study parameters than IS-HVE: *TNC* (t = -15.42, df = 4 p< 0.001); *TNC 60–384* (t = -6.40, df = 4 p = 0.003). DS-HVE was characterized by higher values in both parameters than DS-AE, but the difference was not significant. The same was seen when US-AE was compared to US-HVE. In other words, the aerosol control system had a significant effect only in the case of indirect spray with high-speed turbine, and in that case, HVE was the more efficient method.

### Number concentrations and particle sizes

**Fig 4:**
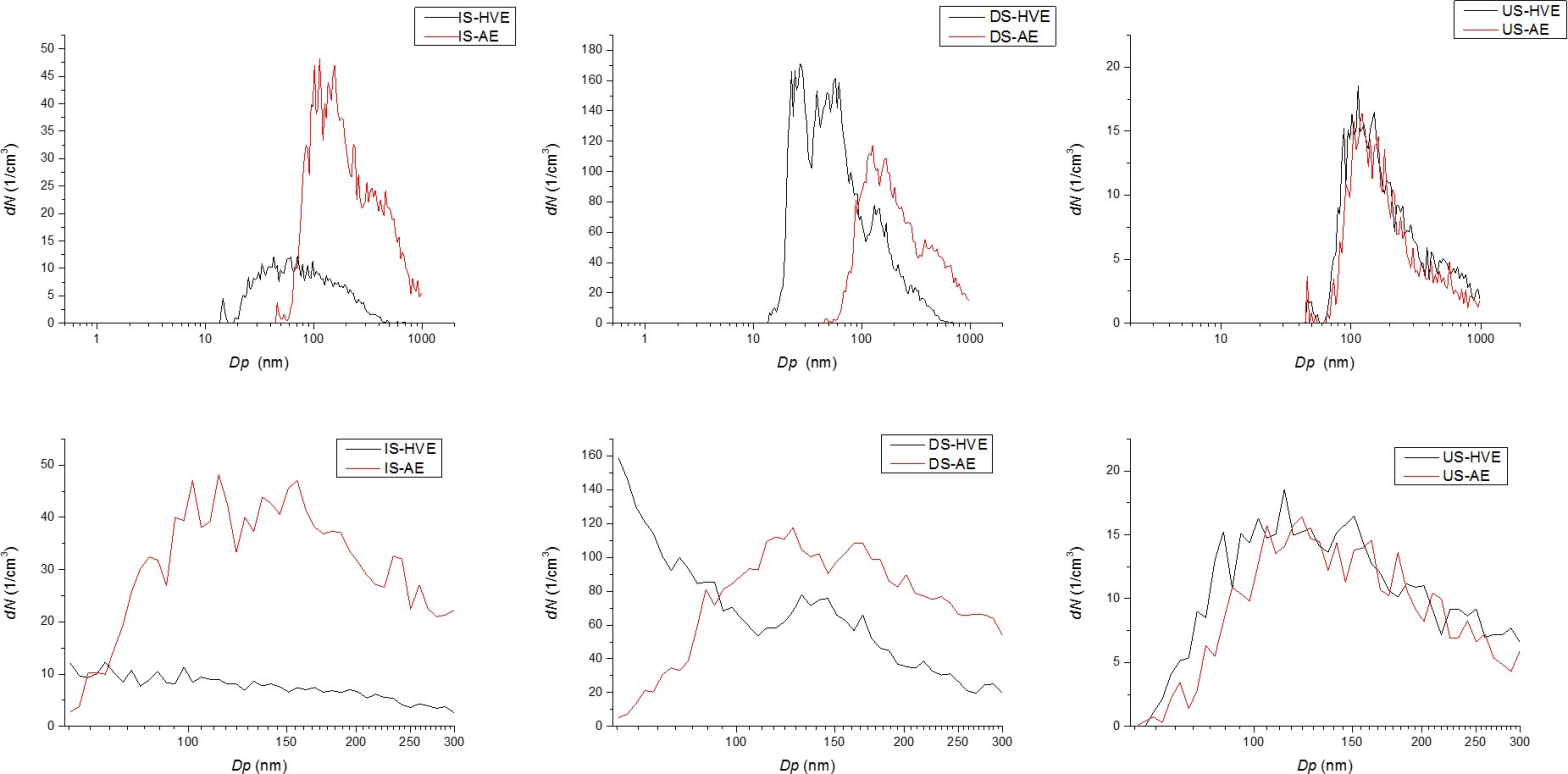
Size distribution of generated water aerosol-the effect of the applied aerosol control system within the groups defined by instrument and spray direction. *dN*: number concentration, *Dp*: particle diameter; top: *TNC* bottom: *TNC 60–384*; IS-HVE: indirect spray with high volume evacuator, IS-AE: indirect spray with aerosol exhaustor, DS-HVE: direct spray with high volume evacuator, DS-AE: direct spray with aerosol exhaustor, US-HVE: ultrasonic scaler with high volume evacuator, US-AE: ultrasonic scaler with aerosol exhaustor.

Fig 4 shows number concentrations and particle sizes. AE and HVE are compared within the groups defined by instrument and spray direction. IS-HVE resulted in moderate number concentration and larger particles, while IS-AE yielded a remarkably higher number concentration in mostly the same size range (for the exact significances see above). As for DS, DS-HVE generated higher number concentrations of smaller particles in comparison with DS-AE. In US, HVE and AE resulted in almost the same outcomes, both in terms of number concentration and particle size. This description fits both the entire range and the 60–384 nm subrange, except for DS, where an interesting difference can be observed between HVE and AE (Fig 4, bottom, middle panel). With HVE, the size distribution of particles is markedly shifted toward the small end of the spectrum: the number concentration of the smallest particles is the highest in the sample, and, progressing toward the high end of the size spectrum, the concentration is steadily on the decline, apart from an insignificant bump between 100 and 150 nm. In contrast, with AE, a near normal size distribution was achieved, with the highest number concentrations toward the middle of the size spectrum.

### Dynamics of aerosol concentration during the airing period

**Fig 5:**
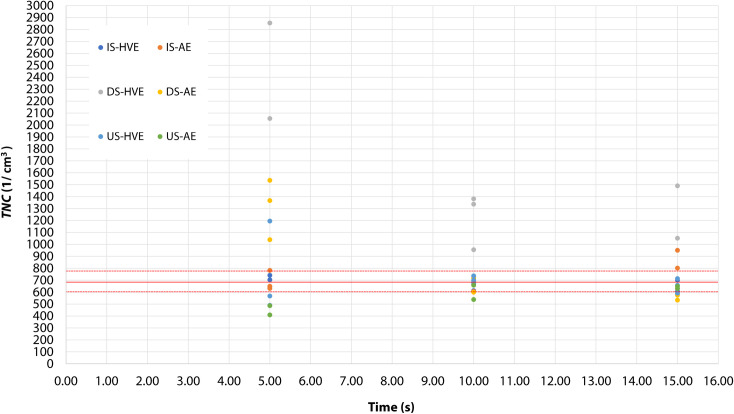
The dynamics of aerosol decay (*TNC*–total number count) during the airing period (3 measurement cycles) after each test measurement (setup). Like the test measurements, airing measurements were carried out in triplicate. Values are given for each scenario at the end of the 5^th^, 10^th^ and 15^th^ minute of airing, corresponding to the measurement cycles. The different setups are represented by colored dots (see legend, the conventions are the same as in Fig 1). Data for all three measurements for all three time points are shown, but please note that the dots may overlap. The solid red line represents the mean baseline level (~697/cm^3^), the dotted lines denote the standard deviation of the baseline mean (see also Table 1).

This is demonstrated in Fig 5 through the dynamics of *TNC* for all setups. The figure shows TNC after 5, 10 and 15 minutes of airing, from three measurements. It is readily observable in the figure that with the applied airing method, a massive drop in *TNC* occurred between 5 and 10 minutes for all setups. In this period, *TNC* dropped back to baseline or below for most of the setups, only DS-HVE remaining above baseline. *TNC* in DS-HVE did not completely return to the baseline even at 15 minutes. Furthermore, at 15 minutes, a minor elevation above the baseline detected in IS-AE again in one case, probably indicating that the concentration of aerosol decayed in a fluctuating manner.

## Discussion

The results of this study allow a quantitative characterization of the generated water aerosol in the whole measuring range of the spectrometer and specifically in the range that is relevant in terms of virus spread. The data do not allow conclusions either regarding the circumstances in which the individual particles were formed or the changes they underwent during their spread. The analysis of such fine changes is beyond the scope of this study, as it is highly unlikely that in the given setting, they could considerably influence the results. However, this a limitation of the study beyond doubt, as are its *in vitro* nature, and the small number of repeated measurements. All these limitations make the study exploratory in nature.

This study sought to determine in a clinically relevant way what aerosol particle concentrations two typical dental instruments featuring air spray generate and how efficiently these concentrations are controlled by two widespread control devices, as such quantitative measurements were lacking. To interpret the results correctly, one must understand that in real-life dentistry, the spray is never exclusively directed inward or outward, rather, the instrument alternates between these positions, and even that with breaks. Direct and indirect mark the endpoints of a spectrum, so the results define the range in which concentration changes may take place during various treatments performed with the studied instruments and controlled with the studied control systems.

Regarding the setups, we hypothesized that both the instrument/ its use and aerosol control would be significant determinants of aerosol concentration.

The results partially support the hypothesis. The type of dental instrument and its way of use was indeed a key factor in aerosol generation. Scaler generated the least aerosol, followed turbine with indirect spray, and turbine with direct spray. This was somewhat surprising, as earlier studies suggested that the ultrasonic scaler was the most problematic instrument in the dental setting in this respect [20].

The applied aerosol control system was not a significant factor in any of the setups, except for indirect high-speed turbine, where HVE was the more efficient method of the two. It must be noted, though, that in the direct turbine setups, AE resulted in markedly lower concentrations. Statistical significance could not be established, but it might easily be a result of the low number of measurements, as the effect is obvious. While the results do not allow strong conclusions about the effect of in-treatment aerosol control, they strengthen the hypothesis that specific types of aerosol control might be better suited for specific settings. This points to the necessity of further studies in this direction.

Regarding the effect of airing between treatments, we hypothesized that a conventional method of airing would be sufficient to reduce aerosol concentration to safe levels in a clinically reasonable timeframe. This was true for all setups, except for DS-HVE (which, as said, is never used exclusively during any treatment). Based on the results, a safety airing period of at least 15 minutes is recommendable between two treatments. By the application of more advanced airing methods (such as a built-in ventilation unit) shorter periods may be achievable.

## Conclusions

Within the limitations of this study, the results clearly show that aerosol generation in the dental operatory largely depends on what instrument is used, and especially in what way. Aerosol generated by the ultrasonic scaler can be controlled more efficiently than aerosol from a high-speed turbine and aerosol from a high-speed turbine reflected from the teeth is controlled more efficiently than aerosol directly from the turbine. Following from this, the dental professional needs to exercise extra care when performing procedures where aerosol might get in the air directly from the high-speed turbine. An example is Class III cavity preparation in the upper front teeth with palatal access, where the spray is directed outward.

As for whether either of the tested aerosol control devices is superior, we cannot offer a firm conclusion, while both devices were safe and efficient. The results suggest that AE might have its advantages in the (least efficiently controllable) direct aerosol situations, but this is only a hypothesis that arose from the data and it needs confirmation.

Finally, regarding the efficiency of airing between two treatments and recommendable airing time, the results suggest that 10 minutes of airing reduces aerosol concentration to a safe level in most typical treatment scenarios (with doors and windows open and using a commercially available standard fan). However, if during the intervention a high amount of aerosol could get in the air directly, it is recommended that at least a 15-minute airing break be observed. Advanced airing methods (e.g. a built-in ventilation unit) may shorten this period.

## Supporting information

S1 File The SPSS spreadsheet used for the analyses. (SAV)
